# Haemoglobin interference and increased sensitivity of fluorimetric assays for quantification of low-parasitaemia *Plasmodium *infected erythrocytes

**DOI:** 10.1186/1475-2875-8-279

**Published:** 2009-12-04

**Authors:** Carlos Moneriz, Patricia Marín-García, José M Bautista, Amalia Diez, Antonio Puyet

**Affiliations:** 1 Departamento de Bioquímica y Biología Molecular IV, Universidad Complutense de Madrid, Facultad de Veterinaria, 28040 Madrid, Spain

## Abstract

**Background:**

Improvements on malarial diagnostic methods are currently needed for the correct detection in low-density *Plasmodium falciparum *infections. Microfluorimetric DNA-based assays have been previously used for evaluation of anti-malarial drug efficacy on *Plasmodium *infected erythrocytes. Several factors affecting the sensitivity of these methods have been evaluated, and tested for the detection and quantification of the parasite in low parasitaemia conditions.

**Methods:**

Parasitaemia was assessed by measuring SYBRGreen I^® ^(SGI) and PicoGreen^® ^(PG) fluorescence of *P. falciparum *Dd2 cultures on human red blood cells. Different modifications of standard methods were tested to improve the detection sensitivity. Calculation of IC_50 _for chloroquine was used to validate the method.

**Results:**

Removal of haemoglobin from infected red-blood cells culture (IRBC) increased considerably the fluorescent signal obtained from both SGI and PG. Detergents used for cell lysis also showed to have an effect on the fluorescent signal. Upon depletion of haemoglobin and detergents the fluorescence emission of SGI and PG increased, respectively, 10- and 60-fold, extending notably the dynamic range of the assay. Under these conditions, a 20-fold higher PG vs. SGI fluorescent signal was observed. The estimated limits of detection and quantification for the PG haemoglobin/detergent-depleted method were 0.2% and 0.7% parasitaemia, respectively, which allow the detection of ~10 parasites per microliter. The method was validated on whole blood-infected samples, displaying similar results as those obtained using IRBC. Removal of white-blood cells prior to the assay allowed to increase the accuracy of the measurement, by reducing the relative uncertainty at the limit of detection from 0.5 to 0.1.

**Conclusion:**

The use of PG microassays on detergent-free, haemoglobin-depleted samples appears as the best choice both for the detection of *Plasmodium *in low-density infections and anti-malarial drugs tests.

## Background

The early detection and monitoring of malarial disease has become a key requirement for the efficient control of *Plasmodium *infections. Asymptomatic individuals in malaria-endemic areas may be infected at sub-microscopic level and still produce gametocytes, contributing to the transmission of the disease [1,2]. Detection of malaria in pregnancy also poses a challenge due to the low parasitaemia in peripheral blood [3]. Malaria diagnosis has relied on either thick blood film microscopy [4] or, more recently, on circulating-antigen (CAg) [5] and PCR-based methods [6-9]. While microscopic methods are simple, they require trained microscopist and do not allow detection at densities below 50 parasites/μL. Real-time PCR showed to be highly sensitive and allows the specific detection of species, but it includes labour-consuming methods such as DNA extraction, and requires expensive equipment and temperature-labile consumables [10].

Highly-sensitive methods are also required for the *in vitro *evaluation of new anti-malarial drugs, prompted by the emergence and spread of parasite resistance [11]. The need to evaluate the anti-malarial activity of new synthetic or natural origin compounds against *Plasmodium falciparum *cultures demands highly sensible and reproducible assays, making indispensable the optimization of existing methods to obtain reliable dose-response curves and adjust the doses of *in vivo *assays. Methods based on enzymatic [12] and radioactive labeled molecules [13] have been used for detecting anti-plasmodial compounds, but they are being progressively replaced by microfluorimetric assays, based on the binding of the asymmetrical cyanine dyes SGI [14-17] or PG [18-20] to DNA. These methods allow evaluation of anti-malarial drug efficacy on erythrocyte cultures, by testing the arrest of the parasite DNA replication. These assays showed to be sensitive, straightforward and rapid, allowing the screening of large number of samples in 96-well format. Different authors claimed the highest sensitivity for both SGI [17] and PG [19] tests.

Both fluorescent dyes display significant structure homologies and have similar molar extinction coefficients, but differ at the group attached to position 2 of the quinolinium ring [21]: SGI has a N-(3-dimethylaminopropyl)-N-propylamino and PG a N-bis-(3-dimethylaminopropyl)-amino residue. The positive charges of PG and SGI are likely contributing to the high binding affinity for dsDNA. SGI carries two positive charges under standard conditions, whereas PG carries three positive charges [21]. These molecules share the property of binding selectively double-stranded DNA (dsDNA) by intercalation, resulting in an increase in fluorescence emission. Although PG reagent is more sensitive for quantitating double-stranded DNA (dsDNA) in solution [19], SGI is frequently used due to its lower cost and higher safety [17].

As reported for PG [20], the use of fluorophores in blood-cultures can be affected by haemoglobin quenching of the fluorescent signal. Due to the wide absorption spectrum of haemoglobin, between 300-800 nm, this molecule could interfere not only with the fluorescence emitted by PG (excitation/emission 390/505 nm) but also with SGI (505/615 nm). In addition, the presence of detergents in the composition of lysis/fluorescent solutions have been reported to have an effect on the detection of DNA with PG [20].

The use of SGI or PG microfluorimetric assays for the detection of *Plasmodium *in clinical isolates may be hampered by the presence of nucleated blood cells in the sample. Aiming to find an optimized assay allowing the highest sensitivity for the detection and quantification of *Plasmodium *DNA in erythrocytes, usable for monitoring both low-parasitaemia malaria in clinical samples and the screening of anti-malarial drugs, a systematic analysis was carried out to asses the effect of detergents, haemoglobin and the presence of white-blood cells in the assay. Hence, a PG-based method is proposed, suitable for detection and quantification of *Plasmodium *in blood and reaching sensitivity near to that reported for PCR-based methods

## Methods

### DNA standard curves in the presence of detergents

SYBRGreen I^® ^(S33102) and PicoGreen^® ^(P7589) were purchased to Invitrogen and diluted as indicated by the manufacturer in TE buffer (10 mM Tris-HCl, 1 mM EDTA, pH 7.5) or TE plus different combinations of saponin and Triton X-100 detergents to obtain a range of concentrations from 1000 ng/mL to 5 ng/mL. Standard curves of DNA were performed by diluting bacteriophage λ DNA, provided at 100 μg/mL in the Quant-iT™ PicoGreen^® ^Kits (Molecular Probes™, Invitrogen) with DNase-free water. One hundred microliters of each mixture were dispensed into black 96-well microplate (Costar^®^), followed by the addition of 100 μl of either PG or SGI. Plates were incubated in the dark at room temperature for 5 minutes and the fluorescence intensity was measured at 485 nm excitation and 528 nm emission using a Perkin Elmer LS-50B Luminescence Spectrophotometer. The mean background reading from the two control wells was subtracted from the test reading to give the final fluorescence measurement. Fluorescence units (arbitrary units ranging from 1 to 1,000 units) was plotted against DNA concentration.

### Haemoglobin effect on fluorescent measurements

Fluorescence measurements in the presence of haemoglobin was carried out by washing uninfected human blood in RPMI1640 medium (final 50% haematocrit) and treated with Lymphoprep™ (AXIS-SHIELD) as indicated by manufacturer to remove most of the WBC (white blood cells). Erythrocytes were resuspended in culture medium [22] to get a haematocrit of 1-5%, and lysed by freeze-thawing. Each sample was spiked with lambda DNA at a final concentration of 1 μg/mL. 100 μL of each mixture were added to 100 μL of either SGI or PG and the fluorescence signal was determined as described above. In addition, the absorption spectrum from red blood cells lysates was evaluated. All measurements were performed in triplicate from three processed samples independently

### Stock culture of *P. falciparum*

Chloroquine resistant strain Dd2 of *P. falciparum *(clone MRA-150, [23] was used for this study. The strain was cultivated *in vitro *as described previously [22]. The culture media consisted of standard RPMI 1640 (Sigma-Aldrich) supplemented with 0.5% ALBUMAX I (Gibco), 100 μM hypoxanthine (Sigma-Aldrich), 25 mM HEPES (Sigma-Aldrich), 12.5 μg/mL gentamicine (Sigma-Aldrich) and 25 mM NaHCO_3 _(Sigma-Aldrich). Erythrocytes were obtained from type A+ human healthy local donors and collected in tubes with citrate-phosphate-dextrose anticoagulant (VACUETTE^®^), and washed as described above. Each culture was started by mixing uninfected and infected erythrocytes to achieve a haematocrit at 1% and incubated in 5% CO2 at 37°C in tissue culture flasks (Iwaki). The progress of growth in the culture was determined by microscopic counting of parasitaemia in thin blood smears with Wright's eosin methylene blue solution (Merck) using the freely available *"Plasmoscore" *software [24]. The cultures were synchronized by incubation with sorbitol as described [25].

### Determination of IC_50_

Synchronized rings from stock cultures were used to test 5 μM to 1 nM serial dilutions of chloroquine in 96-well culture microplates. Thus, 150 μL of parasites at 2% haematocrit and 1% parasitaemia were allowed to grow for 48-hour in 5% CO_2 _at 37°C. Parasites were then centrifuged at 600 × g for 10 minutes and the re-suspended in saponin to lyse the erythrocytes. The pellet was washed to remove haemoglobin as described [20]: 150 μL of each sample were dispensed in 96-well microplates, which were centrifuged at 600 × g for 10 minutes. The supernatant was removed and the cells were re-suspended in saponin (0.15%, w/v in phosphate-buffered saline (PBS) to lyse the erythrocytes and release the malaria parasites. The microplates were immediately centrifuged at 600 × g for 10 minutes, and the supernatant was removed. To eliminate all traces of haemoglobin the pellet was washed by the addition of 200 μL of PBS followed by centrifugation at 600 × g. The washing step was repeated twice to ensure complete removal of haemoglobin. Finally, pellets were re-suspended in 100 μL of PBS. 100 μL of either PG or SGI diluted in TE were added to each well. Then, the plates were incubated for 30-60 minutes in the dark. The fluorescence signal was determined as described above. Fluorescence Unit (AU) was calculated by subtracting the control negative (fluorescence from uninfected erythrocytes) [13]. AU was converted to percentage (%) of survival in the fluorimetric drug susceptibility test by the following equation:

Where, *Fd *is the fluorescence from drug treated infected erythrocytes, *Fcp *is the fluorescence control positive: fluorescence from non-treated infected erythrocytes, and *Fcn *is fluorescence control negative: fluorescence from uninfected erythrocytes. The logarithm of the chloroquine concentration was plotted against the corresponding percentage (%) of survival, and the 50% inhibitory concentration (IC_50_) was calculated by non-linear regression. All measurements were performed in triplicate from two processed samples independently. The curve fitting was performed using Sigmaplot^©^10 from Systat Software, Inc.

### Correlation of parasitaemia and fluorescence

A serial dilution of the synchronized cultures of *P. falciparum *(mature ring stage) was performed with non-parasitized erythrocytes and complete medium to yield a haematocrit of 2% and parasitaemia levels ranging from 0.05% to 15%. Haemoglobin-depleted samples were obtained as indicated above. In these assays, 100 μL of either SGI or PG were added to each microplate well and the fluorescence signal was determined. To analyse the effect of TritonX-100, a set of haemoglobin-depleted samples were mixed with SGI or PG plus TritonX-100 (2% v:v final concentration). Control samples without haemoglobin removal were analysed in parallel. In this case, the parasitized erythrocytes were lysed by using the lysis/fluorescence-mix, consisting of either SGI, 0.008% saponin and 0.08% Triton X-100, or PG with 2% Triton X-100. Fluorescence readings were plotted against parasitaemia.

Assays on whole blood were carried out by diluting a sorbitol-synchronized culture with a donor peripheral blood to achieve different levels of parasitaemia from 15% to 0.05%. Two series of dilutions were performed: the first series was prepared at 50% haematocrit and 400 μL were added to 200 μL Lymphoprep™ to remove WBC. The resulting sediment was washed and diluted with phosphate buffered saline to obtain 2% final haematocrit. The second series was directly diluted to 2% haematocrit without WBC removal. Haemoglobin was depleted both from Lymphoprep-treated and non-treated samples, and PG-DNA fluorescence was determined as above.

All measurements were performed in triplicate from three processed samples independently. The relative uncertainty (relative error) was calculated as the standard deviation divided by the mean. The limit of detection (LOD) was established as the lowest parasitaemia yielding fluorescent signal significantly different from the blank control. The limits quantification (LOQ) corresponds to the lowest parasitaemia that can be quantified with acceptable accuracy and precision. LOD and LOQ were calculated as indicated by the International Conference on Harmonization (ICH) guidelines [26] by using:

Where s_B _is the standard deviation of the blank sample, *m *is the slope of the calibration curve and *t *= 3.3 is the Student's t for a 95% confidence level. Statistics and curve fitting was performed using Sigmaplot^©^10 from Systat Software, Inc.

## Results

### Effect of detergents on SGI and PG DNA measurements

**Figure 1:**
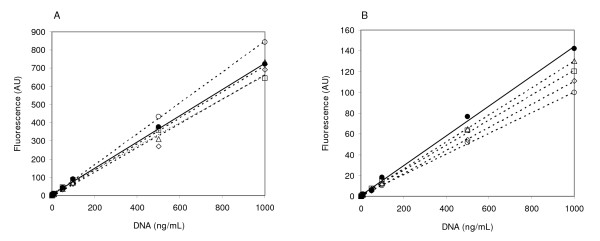
**DNA titration using SYBR^®^Green I and PicoGreen^® ^dyes**. PicoGreen^® ^fluorescence (A) and SYBR^®^Green I fluorescence (B) with bacteriophage λ DNA in absence (black circle, solid line), or presence (dotted lines) of different amounts of detergents: saponin 0.008% + Triton X-100 0.08% (white triangle); saponin 0.008% (white square); Triton X-100 0.08% (white diamonds); Triton X-100 2% (white circle). Background fluorescence, defined as fluorescence detected in the absence of DNA, was subtracted from each data point. Fluorescence is measured as arbitrary units (AU). Data show average from three replicate experiments. Error bars indicate standard deviations. Lines were calculated by linear regression; r^2 ^>0.99.

Several fluorimetric-based protocols make use of different combinations of saponin and TritonX-100 as detergents for the combined erythrocyte lysis/fluorescence detection in parasitaemia measurements. To ascertain the effect of these detergents in SGI and PG assays, DNA standard curves were prepared in the presence of different detergents currently used in lysis/fluorescent mixes. The results showed a direct relationship between DNA concentration and fluorescence (Figure 1). Higher signal was detected for PG (706 ± 33 AU per 1 μg/mL) as compared to SGI (141 ± 40 AU per 1 μg/mL) in TE. The presence of detergents changed the slope of the standard curves for both dyes. All combinations tested generated a decrease in the fluorescent signal, with the exception of PG 2% Triton X-100 which displayed a slight increment. The mixture of 0.008% saponin and 0.08% Triton X-100 appears to have a smaller effect of both dyes (<10% in SGI, <2.5% for PG), while these detergents added separately showed intermediate results.

### Effect of haemoglobin on fluorescence

The effect of haemoglobin on fluorescent readings was tested by mixing different amounts of lysed erythrocytes (1-5% haematocrit) with a fixed amount of λ DNA. Erythrocytes were lysed by freeze-thawing to avoid the use of detergents which, as shown above, might interfere with the fluorescence measurements.

**Figure 2:**
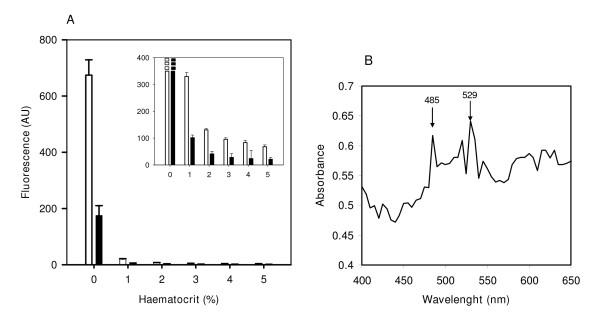
**Effect of haemoglobin on fluorescence emission**. **A) **Fluorescence emission of SYBR^®^Green I (solid bars) and PicoGreen^® ^(empty bars) of 1 μg/mL DNA, (485/2.5 nm excitation, 528/2.5 nm emission) in the presence of different amounts of red blood cells. The inset shows the fluorescence signal at 485/15 nm excitation and 528/15 nm emission. Discontinuous bars indicate off-scale fluorescent reading. Fluorescence at 0% haematocrit was obtained from a 1 μg/mL solution of λDNA in culture medium. **B) **Absorption spectrum of red blood cell lysates.

The fluorescence emission of both SGI- and PG-DNA adducts, shown in Figure 2A, was highly quenched in presence of red cells. Control samples, containing DNA without haemoglobin, rendered fluorescence readings around 30-60 fold as compared with samples containing blood, suggesting a heavy interference of haemoglobin. Detection of fluorescence in presence of blood, even at the lowest content, was only possible by opening the optical slit of the detector to 15 nm. Under these conditions the fluorescence emission from 1 μg/mL DNA samples in 0% haematocrit was rendered off scale.

As shown in Figure 2B the absorption spectrum of red blood cell lysates revealed the existence of two peaks of maxima absorption at 485 nm and 529 nm, which overlap with the excitation/emission wavelengths of SGI and PG, and may therefore explain the quenching of haemoglobin on the fluorescent readings.

### Calculation of IC_50 _for chloroquine

**Figure 3:**
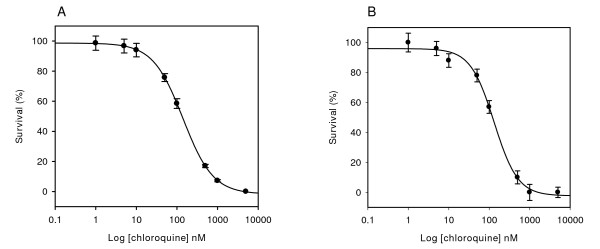
**Chloroquine dose-response curve**. Chloroquine responses of *P. falciparum *in erythrocyte cultures obtained using PG (A) and SGI (B), after removal of haemoglobin/detergents. Sigmoid graphs show the survival of *P. falciparum *Dd2 at different concentrations of drug. Bars indicate the standard errors of the mean for two independently processed samples. The calculated IC_50 _values were 136.2 ± 28 nM for PG and 118 ± 15 nM for SGI.

To test if the signal increase observed after depletion of haemoglobin may lead to changes in the calculated IC_50 _values of current anti-malarial drugs, dose-response curves for chloroquine were obtained using either PG or SGI. No significant differences in the IC_50 _values were observed for chloroquine in fluorimetric anti-malarial drug assays run in parallel (p > 0.05) (Figure 3). The IC_50 _for chloroquine against strain Dd2 of *P. falciparum *was 136.2 ± 28 nM when using PG and 118 ± 15 nM for SGI. These values were similar to those reported by other authors using different techniques [15,27,28]. Chloroquine is a known DNA intercalating agent [29,30], and it has been shown that it may lead to 23% PG exclusion in the range of 0.5 to 1 mM [31]. The possible effect of the drug on the binding of the fluorescent dye to DNA at concentrations used in the IC50 assay was tested by comparison of the DNA-PG calibration curves in the presence of chloroquine at 1 μM and 5 μM. No significant differences in the slope or intercept were observed as compared to the control curve without drug (p > 0.05, data not shown), suggesting that chloroquine do not interfere with PG binding to DNA well above the highest concentration used.

### Fluorescent signal as measurement of *Plasmodium *parasitaemia

As shown above, the choice of DNA-fluorescent dye and the removal of haemoglobin before fluorescent readings may enhance the sensitivity of the assay. In addition, the presence of 2% Triton X-100 showed an increase in the fluorescent signal of PG-DNA adducts, while other detergent formulations may reduce that signal (Figure 1). To analyse the effect of haemoglobin depletion and the addition of 2% Triton X-100 on the quantification of *Plasmodium *parasitaemia, the fluorescence obtained in cultures with or without haemoglobin removal was compared. While samples containing haemoglobin were lysed by adding a fluorescent mix containing detergents, haemoglobin-depleted samples were first lysed with saponin, washed and resuspended in PBS, and the fluorescent mix was added without detergents, avoiding thus any interference due to these compounds. The effect of Triton X-100 was tested only on haemoglobin-depleted samples, by mixing PG or SGI with TritonX-100 2%.

**Figure 4:**
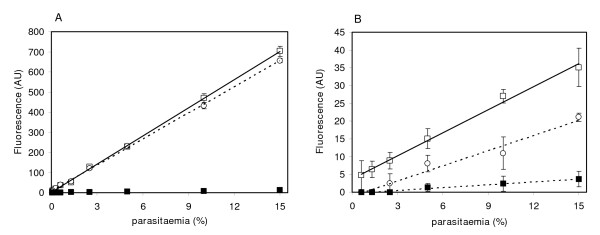
**Correlation between parasitaemia and fluorescence emission**. PG (A) and SGI (B) fluorescence detected in *Plasmodium falciparum *infected erythrocytes at different parasitaemias. A serial fold dilution of synchronized infected culture (15% mature ring stage) with non-infected erythrocytes was used. Results are shown for haemoglobin non-depleted samples (black square), and haemoglobin depleted samples in the absence (white square) or presence (white circle) of 2% Triton X-100. Bars indicate the standard deviation of the mean from three independently processed samples.

The results, shown in the Figure 4, confirm the linear correlation between the percentage of infected red blood cells and the fluorescence. As expected, PG exhibited much higher fluorescence emission than SGI (20-fold at 15% parasitaemia). SGI results showed a higher interference of TritonX-100 by decreasing both the slope (2.2 versus 1.4 AU/%parasitaemia) and the correlation of the linear response (*r*^*2 *^> 0.99 to *r*^*2 *^> 0.97). Best results were obtained with PG in samples devoid of haemoglobin (*r*^*2 *^> 0.99, *m *= 46.6): for a 15% parasitaemia culture, fluorescent readings were in the range of 650-700 vs. 11 (arbitrary fluorescence units) in non-depleted haemoglobin. The presence of 2% TritonX-100 showed a much smaller effect, with no clear gain or loss on the signal.

The fluorescence of highly diluted samples of a *P. falciparum *culture using SGI or PG under these conditions (no haemoglobin, no detergent) is shown in Table 1. While SGI was not able to detect a parasite load below 0.6%, PG yielded fluorescent readings down to 0.05% and showed a 7-fold higher signal than SGI at 0.6% parasitaemia, leading to a similar reduction of the calculated LOD from 1.4% to 0.2%. Similarly, a 6-fold reduction of the LOQ was observed when using PG as compared to SGI.

**Table 1:** Fluorescence of PG and SGI - DNA adducts on *Plasmodium *infected erythrocytes at low parasitaemia after removal of haemoglobin and detergents

	Fluorescence units (AU)			
	
	**SYBRGreen I**^**®**^	**PicoGreen**^**®**^		
		
% parasitaemia	IRBC	IRBC	IRBC-WBC	IRBC-WBC/W
5	15 ± 3	229 ± 12	243 ± 4	279 ± 8
2.5	9 ± 2	127 ± 7	141 ± 8	161 ± 7
1.3	6 ± 2	52 ± 1	83 ± 9	80 ± 4
0.6	5 ± 4	36 ± 3	36 ± 11	45 ± 2
0.3	ND^1^	20 ± 5	20 ± 8	31 ± 4
0.1	ND	11 ± 3	11 ± 3	14 ± 2
0.05	ND	5 ± 4	ND	7 ± 3

**Limits of detection and quantification (parasitaemia %)**				

LOD	1.37	0.23	0.21	0.24
LOQ	4.14	0.71	0.74	0.64

IRBC = Infected red-blood cells; IRBC-WBC = Infected red-blood cells whithout white blood cells removal; IRBC-WBC/W = Infected red-blood cells after white blood cells removal; ND = not detected.

**Figure 5:**
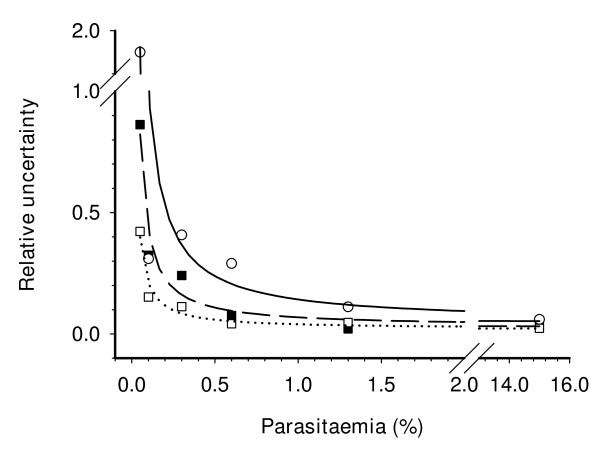
**Relative uncertainty of fluorescent measurements of PG-DNA adducts in low parasitaemia samples**. Plot of the relative uncertainty calculated form the fluorescence readings at different parasitaemias shown in the Table 1. Results are displayed for infected red-cell cultures (IRBC, black square, dashed line), infected red-cell cultures diluted with peripheral blood (IRBC+WBC, white circle, solid line), and infected red-cell cultures diluted with peripheral blood and depleted of white-cells (IRBC+WBC/W, white square, dotted line). All samples were processed for removal of haemoglobin and detergents. Curves fitted using inverse first order polynomial equation, *r*^*2*^> 0.97.

Once established the PG haemoglobin/detergent-depleted method as the most appropriate for low-parasitaemia samples, its capability for detection and quantification of *Plasmodium *in whole blood samples, as those coming from clinical isolates, was tested. A synchronized *P. falciparum *culture was diluted with human peripheral blood to obtain parasitaemia ranging from 15% to 0.05% (IRBC+WBC samples). Each dilution was split in two and one of them was treated with Lymphoprep™ to remove WBC (IRBC+WBC/W). Haemoglobin was depleted from both samples and fluorescence was measured with PG. The results showed a good correlation of fluorescence versus parasitaemia (*r*^*2 *^> 0.99) in both conditions. Data at parasitaemia in the range of 5% to 0.05% are shown in the Table 1, indicated that the method can detect down to 0.2% parasitaemia even in the presence of WBC DNA, although the high variability observed in these samples may compromise the accuracy of results. To solve this problem, an additional wash step was included in the protocol to remove WBC prior to the fluorescent reading. The Figure 5 shows a comparison of the relative uncertainty of the measurements obtained from IRBC, IRBC+WBC and IRBC+WBC/W samples at low parasitaemia. While the relative uncertainties were very low (<0.15) for parasitaemias >1.5% in all samples, only IRBC and IRBC+WBC/W samples showed relative uncertainties <0.3 at the LOD and LOQ values: Removal of WBC reduced the relative error from 0.5 to 0.12 at 0.2% parasitaemia, and from 0.2 to 0.04 at 0.6% parasitaemia. These results suggest that white cell removal should be performed to achieve the highest accuracy at the LOD and LOQ range.

The possibility of increasing the sensitivity by using higher haematocrit was also explored by measuring fluorescence from IRBC+WBC/W samples at 5% and 10% haematocrit. The results (not shown) displayed a non-linear correlation of fluorescence to parasitaemia, which could be associated to the accumulation of cell-debris after lysis interfering with the fluorescence measurement.

## Discussion

Monitoring pathogen DNA by fluorimetric methods appears to be a suitable technique for the determination of parasitaemia in infected cells. The system may be particularly effective for the high-throughput screening of potential anti-parasitic drugs, as their effect on the arrest of pathogen DNA replication can be easily detected and quantified. These methods depend largely on the sensitivity and dynamic range of the fluorescent emission obtained with DNA intercalating agents. Highly sensitive fluorescent dyes should allow the use of small culture volumes, the monitoring of low parasitaemias and the high-throughput assays of anti-malarial drugs. In this study it is shown that PG is 5-fold more sensitive than SGI on purified DNA samples. Haemoglobin has been reported to drastically quench the fluorescent signal of DNA-PG adducts [20], while other authors do not observed significant effects of haemoglobin under the same conditions [19]. In this work, such interference has been quantified, showing that in DNA/lysed red blood cell mixtures (2% haematocrit), the fluorescent signal was quenched to 1% (PG) or 2% (SGI) of the purified DNA reading. The absorption spectrum of haemoglobin shows two peaks at 485 and 529 nm which overlap almost exactly the excitation/emission wavelengths for SGI [17,21] and PG [32].

Detergents are used routinely in the preparation of lysis/fluorescent mixes in fluorimetric method for detection of anti-malarial drugs [17,19,20]. The use of diluted detergents may facilitate the adequate dye-nucleic acid interaction and detection of fluorescence. However, different results have been reported on the effect of detergents: while PG appeared not to be affected by TritonX-100 up to 2% [20], lower fluorescent signal was detected in SGI on 0.08% TritonX-100 [17]. The results showed in this work suggest that, in general, the presence of Triton X-100 and other detergents decrease the fluorescent signal of both dye-DNA adducts. Although the presence of 2% TritonX-100 increases slightly the fluorescence of PG on λ DNA, no clear differences were observed on cultures of *Plasmodium *in erythrocytes. Consequently, and as a general guideline, it would be desirable to remove all detergents prior to the measurement of fluorescence.

In order to both remove haemoglobin and prevent the presence of detergents in the fluorimetric assay, *Plasmodium *infected erythrocytes were disrupted by using saponin, followed by several washes for removing haemoglobin and detergent, and no additional detergents were used in the fluorescent dye solutions. Using this approach, 10-fold and 60-fold increases in sensitivity were obtained for SGI and PG respectively. In addition, and differently from other methods published, the proposed protocol was performed in a single 96-well microplate, allowing both parasite cultivation and fluorescent reading in the same plate, circumventing any culture transfer which may increase the variability of results. As shown in this work, the calculation of IC_50 _was not affected by the choice of DNA fluorescent dye or the presence of haemoglobin/detergents in the sample, suggesting that low sensitivity is not critical for estimation of the concentration yielding a 50% parasite survival. However, the optimization of the microfluorimetric method to increase sensitivity should be critical for other applications where microscopic methods are not effective, such as the monitoring of sub-microscopic infections the early stages of disease or in pregnancy and estimation the efficiency of vaccines. Microfluorimetric methods had not been tested previously on whole blood samples. Here, it is shown that a PG haemoglobin/detergent-depleted microfluorimetric assay allows to decrease the LOD to 0.2% parasitaemia on whole blood samples. The relative high dispersion of the fluorescence readings observed at this parasitaemia was reduced by adding a WBC wash prior to the haemoglobin removal. This additional step can be easily performed by any WBC removal protocol adapted to microplate format. Although Lymphoprep was used here for WBC removal, other methods are currently available and could be used for elimination of non-erythrocytic cells. In a recent report, a CF11 cellulose filter was shown to be particularly efficient for removal of leukocyte and platelet cells from whole blood [33]. The use of this method could further improve the sensitivity of the assay in clinical samples.

The 0.2% parasitaemia LOD established for this protocol corresponds to 10 parasites per μL. Thick blood film examination by an experienced microscopist should detect 50 parasites/μL [5], although routine laboratory diagnosis achieves a much lower sensitivity of ~500 parasites/μL [34]. Nested-PCR techniques have been reported to achieve <10 parasites/μL [35]. Quantitative real-time PCR may reach even lower thresholds ranging from 0.3 to 3 parasites/μL depending on the *Plasmodium *species [36]. High rates of sensitivity and specificity have been reported for real-time PCR on clinical isolates [10]. PCR methods have, however, their own limitations, as amplicon numbers may not reflect the number of parasitized cells: the presence of multiple copies of the target DNA, multinucleate schizonts, and DNA released from lysing cells may cause overestimation of the actual parasitaemia [37,38]. Relative high-cost and the need to operate in environments maintaining a cold chain also hinders the use of these methods in most malaria endemic areas. Due to these limitations, the use of more than one established molecular method as gold standard to assess novel malaria diagnostic kits has been proposed [10].

While the proposed protocol allows to diagnose the presence of the parasite in infections at 0.2% parasitaemia, quantitative results can be accurately obtained from 0.6% parasitaemia samples. This capability can be useful for the monitoring of residual infections or for the screening of new anti-malarial compounds.

This comparative study has shown that uncomplicated methodological improvements of fluorimetric tests, such as haemoglobin and detergent depletion on PG-based assays, can enhance their sensitivity to be comparable to that obtained by other molecular methods, preserving the low work load and quickness intrinsic to these methodologies. The additional removal of WBC extends the usefulness of the modified fluorimetric method to the diagnosis and monitoring of *Plasmodium *infections in clinical applications. This assay might be easily standardized and automated for large-scale analysis, facilitating monitoring of low parasitaemias in infected red-cells, as well as for *in vitro *screening of anti-malarial drugs.

## Conclusion

A modified PicoGreen microfluorimetric method allows to reach sensitivity on parasitaemia assays similar to those obtained with PCR-based methods. The proposed approach would be best suitable for epidemiological studies allowing parasite detection on sub-microscopic and/or asymptomatic infections in endemic countries, as well as for large-scale trials on new anti-malarial drugs.

## Competing interests

The authors declare that they have no competing interests.

## Authors' contributions

CM and PM carried out the laboratory work, contributed in the analysis of data and helped to draft the manuscript. JMB, AD and AP participated in the analysis and interpretation of the data, and wrote the manuscript. AD and AP conceived and coordinated the study. All authors read and approved the final manuscript.
